# The mycosporine-like amino acids porphyra-334 and shinorine are antioxidants and direct antagonists of Keap1-Nrf2 binding

**DOI:** 10.1016/j.biochi.2018.07.020

**Published:** 2018-11

**Authors:** Ranko Gacesa, Karl P. Lawrence, Nikolaos D. Georgakopoulos, Kazuo Yabe, Walter C. Dunlap, David J. Barlow, Geoffrey Wells, Antony R. Young, Paul F. Long

**Affiliations:** a Faculty of Life Sciences & Medicine, King's College London, United Kingdom; b School of Pharmacy, University College London, United Kingdom; c Chemical Laboratory, Hokkaido University of Education, Japan

**Keywords:** Mycosporine-like amino acids, Nrf2, Antioxidants, Natural products, Oxidative stress

## Abstract

Mycosporine-like amino acids (MAAs) are UVR-absorbing metabolites typically produced by cyanobacteria and marine algae, but their properties are not limited to direct sun screening protection. Herein, we examine the antioxidant activities of porphyra-334 and shinorine and demonstrate that these MAAs are prospective activators of the cytoprotective Keap1-Nrf2 pathway. The ability of porphyra-334 and shinorine to bind with Keap1 was determined using fluorescence polarization (FP) and thermal shift assays to detect Keap1 receptor antagonism. Concomitantly, the ability of porphyra-334 and shinorine to dissociate Nrf2 from Keap1 was confirmed also by measurement of increased mRNA expression of Nrf2 targeted genes encoding oxidative stress defense proteins in primary skin fibroblasts prior and post UVR exposure. Surprisingly, enhanced transcriptional regulation was only promoted by MAAs in cells after exposure to UVR-induced oxidative stress. Furthermore, the *in-vitro* antioxidant activities of porphyra-334 and shinorine determined by the DPPH free-radical quenching assay were low in comparison to ascorbic acid. However, their antioxidant capacity determined by the ORAC assay to quench free radicals via hydrogen atom transfer is substantial. Hence, the dual nature of MAAs to provide antioxidant protection may offer a prospective chemotherapeutic strategy to prevent or retard the progression of multiple degenerative disorders of ageing.

## Highlights

• Mycosporine like amino acids (MAAs) are natural UVR filters found predominantly in marine species.
• MAAs act as antagonists of Keap1-Nrf2 binding, and as chemical quenchers.
• MAAs activated Nrf2-regulated genes, but only after UV irradiation.
• This also led to decreased expression of MMP1, a collagenase linked to photoageing.
• MAAs offers new insight into how cells can manage UV-induced oxidative stress.

## Graphical abstract

**Figure:**
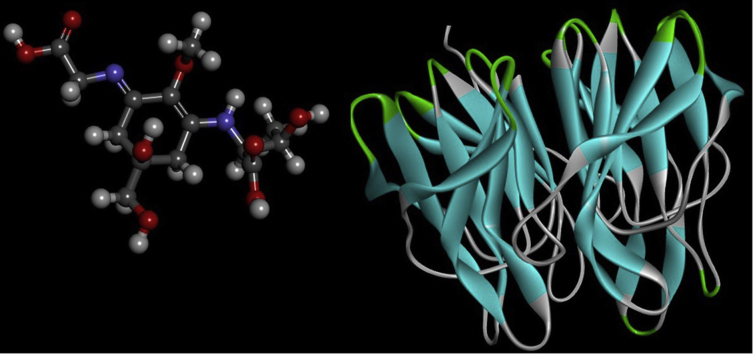

## 1 Introduction

**Fig. 1:**
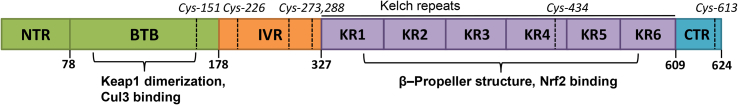
**Illustration of the Kelch-like ECH-associated protein 1 (Keap1).** The Broad-complex, Tramtrack and Bric-a-Brac (BTB) domain, coloured green, is responsible for the formation of the Keap1 dimer and for Cul3 binding; the Kelch-repeat (KR) domain, forms a six-bladed β-propeller structure with DLG and ETGE motifs that bind with the Neh2 domain of Nrf2 [6,10,13]. The Intervening (IVR) domain is comprised of amino acid residues between BTB and Kelch repeats. Cysteine residues that function as redox sensors are denoted in the above illustration.

The Kelch-like ECH-associated protein 1 (Keap1) is an actin bound homodimer that functions as a primary sensor of intracellular reduction-oxidation (redox) state regulation by controlling the activity of the master transcription nuclear factor erythroid 2–related factor 2 protein (Nrf2), which regulates the transcription of a large number of genes under control of the cis-acting enhancer termed the antioxidant response element (ARE) [1,2]. The human Keap1 monomer is a 69.7 kDa protein composed of 625 amino acids that is divided into five distinct domains (Fig. 1). The Kelch-repeat domain consists of six repeating motifs (KR1–KR6) that form a six-bladed *β*-propeller structure at which Keap1 binds to the Neh2 domain of Nrf2 [3]. Under basal conditions, Nrf2 is targeted for ubiquitination and rapid 26S proteasomal degradation by Keap1 BTB domain bound Cullin3-Rbx1 E3 ubiquitin ligase (CRL^Keap1^) [[4], [5], [6]]. This turnover of Nrf2 prevents unnecessary expression of genes under Nrf2 transcriptional regulation [4]. During conditions of oxidative stress, the ubiquitination and degradation of Nrf2 by CRL^Keap1^ is disrupted by Nrf2-Keap1 dissociation. Two separate models have been proposed for this dissociation: the “conformation cycling model” [5] and the “hinge and latch model” [6]. In the conformation cycling model, it is proposed that, in the presence of cellular oxidants and exogenous electrophiles, covalent modification of Cys^151^ in the BTB domain of Keap1 causes conformational changes that prevent ubiquitination of Nrf2 by the CRL^Keap1^ protein complex [6,7]. In the hinge and latch model, the Nrf2-Keap1 interaction is mediated by a high-affinity ETGE motif in the Neh2 domain of Nrf2, which functions as a “hinge” by stabilising Nrf2 binding to the Kelch domain in the Keap1 dimer. A low-affinity DLG motif in the Neh2 domain of Nrf2 functions as the “latch” by locking or unlocking the binding position of Nrf2, depending on the redox state of the cell. Under basal conditions, the DLG motif locks the Neh2 domain in the correct position to enable ubiquitination of Nrf2 [8,9]. However, the IVR domain of Keap1 is cysteine rich and these residues are sensitive to oxidation [[10], [11], [12]]. During conditions of oxidative stress, these cysteine residues become oxidized and although the Nrf2 “latch” remains in a closed position with Nrf2 still bound to Keap1 through both its ETGE and DLG motifs, there is a conformational change in Keap1 such that Nrf2 is no longer correctly aligned with the CRL^Keap1^ complex and Nrf2 ubiquitination is thus disrupted [7]. As a consequence, Nrf2 is spared degradation at the proteosome, and newly translated Nrf2 proteins accumulate in the cell. Free Nrf2 then translocates to the nucleus where it forms a complex with small Maf proteins and interacts with the promoter region of the ARE to initiate the transcription of genes encoding a vast arsenal of proteins that protect against toxic contamination and regulate metabolic redox homeostasis [4,6,11].

Since oxidative stress has been implicated in numerous human diseases, the Keap1–Nrf2 protein-protein interaction (PPI) has become an important target for the potential development of therapeutic and chemopreventive agents [9]. Numerous compounds have been examined for their ability to induce Nrf2-dependent gene expression, including those of natural origin (e.g., curcumin, sulforaphane) and others that are synthetic (e.g., bardoxolone methyl, oltipraz). Most of these Nrf2 activators are electrophiles that covalently modify the sulfhydryl groups of Keap1 cysteine residues disrupting the ubiquitination and subsequent degradation of Nrf2 [13]. These electrophilic inhibitors lack selectivity and thus increase the risk of “off-target” toxic effects due to indiscriminate reactions with cysteine residues of other cellular proteins. Accordingly, the discovery of direct, non-reactive, small molecule inhibitors of the Keap1–Nrf2 PPI appears to be the most promising strategy for Nrf2 activation to decrease the possibility of “off-target” toxic effects [14,15].

**Fig. 2:**
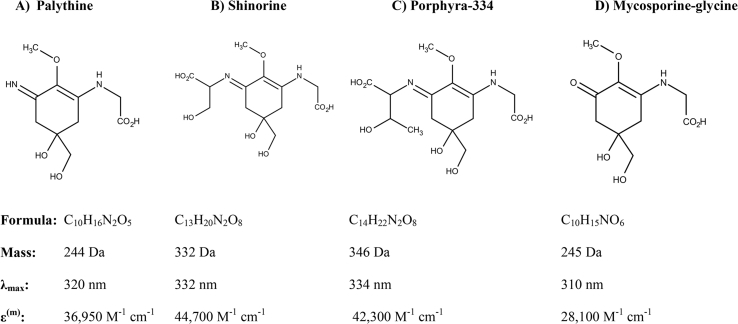
**Structures and biophysical characteristics of MAAs tested in this study or of relevance for discussion in the text.** Figure shows chemical structures of the mycosporine-like amino acids, palythine, shinorine, porphyra-334 and mycosporine-glycine. The molecular formula, molecular mass, maximum wavelength of absorbance and absorbance coefficients are given for each MAA.

Mycosporine-like amino acids (MAAs) are small secondary metabolites commonly produced by marine algae and seaweeds that reside in shallow-water environments and are typically exposed to high levels of solar UVR (∼295–400 nm) [16]. MAAs are found also in the tissues of some marine vertebrates and invertebrates, such as fish and krill, that occur by dietary accumulation from the marine food-chain [17,18]. MAAs absorb UVR typically between 310 and 340 nm, enabling MAAs to protect cells from solar UVR-induced damage. Yet, MAAs are multifunctional metabolites that protect also against free-radical damage and boost cellular tolerance to desiccation, hyper-salinity and heat stress [16,19]; there are more than 20 known MAAs in this class of natural metabolites (Fig. 2).

Previously, utilising protein modelling and virtual screening methods, we had predicted the potential for Nrf2 activation by competitive inhibition of its binding to Keap1, specifically by certain UVR-protective MAAs [20]. Here we provide *in vitro* empirical evidence to confirm our *in silico* predictions that porphyra-334 (the principal MAA of *Porphyra yezoensis* – Japanese seaweed “nori”, Fig. 2c) and shinorine (from *Gloiopeltis furcata* – Japanese seaweed “fukuro-funori”, Fig. 2b) may exert a cytoprotective function by specific, non-reactive binding to the Kelch-repeat domain of Keap1. Additionally, these MAAs are shown to have intrinsic antioxidant activity by quenching free oxygen radicals through hydrogen atom transfer.

## 2 Materials & methods

### 2.1 Materials

All chemicals were purchased from commercial suppliers and used without further purification.

Ascorbic acid, tBHQ, caffeic acid and DMSO were purchased from Sigma-Aldrich (Gillingham, Dorset, UK). Curcumin, DPPH, quercetin, and EGCG were purchased from Insight Biotechnology Ltd (Wembley, Middlesex, UK). Chlorogenic acid, trans-resveratrol and sulforaphane were purchased from Cambridge Bioscience Ltd (Cambridge, UK). Purified MAA compounds porphyra-334 and shinorine were kind gifts from Prof Kazuo Yabe [21,22]. The peptides FITC-(βAla)-DEETGEF-OH and Ac-DEETGEF-OH were synthesised using Fmoc solid phase peptide synthesis as described previously [23].

### 2.2 Fluorescence polarization (FP) assay

The FP assay was carried out as previously described [23]. Briefly, a solution of fluorescent peptide (FITC-(βAla)-DEETGEF-OH, 1 nM) and Keap1 Kelch domain (200 nM) in DPBS, pH 7.4 were mixed in an untreated black 96 well plate (Corning) with varying concentrations of test compounds up to 100 μM (final DMSO concentration 11%, final volume 100 μL) and incubated for 1 h at room temperature in the dark. FP was measured using a PerkinElmer EnVision™ Multilabel Plate Reader. All measurements were recorded in triplicate. The normalised data were fitted to a standard dose-response equation by non-linear regression using Origin Pro software (OriginLab) to determine IC_50_ values.

### 2.3 Thermal shift assay

The thermal shift assay was carried out as previously described [24]. Briefly, a solution of the detection dye SYPRO^®^ orange (5X) and Keap1 Kelch domain protein (5 μM) in DPBS, pH 7.4 were mixed in a MicroAmp^®^ Optical 96-well reaction plate (ThermoFisher) with varying concentrations of test compounds up to 100 μM (final DMSO concentration 10%, final volume 40 μL). The plate was sealed using an optical adhesive cover and wrapped with aluminium foil to protect the dye from light. The plate was then transferred to a plate centrifuge and spun briefly (200×*g*, 1 min, at room temperature) to remove any air bubbles and to collect the reaction mixture at the bottom of the wells. The plate was incubated for 1 h at room temperature and then placed into an Applied Biosystems 7500 Real-Time PCR machine and heated using a standard protein melting protocol [25]. The fluorescence intensity was recorded with excitation at 465 nm and emission measured at 580 nm during a temperature scan from 25 °C to 95 °C with a temperature ramping rate of 1 °C/min. All measurements were performed in triplicate. The raw data were exported to MS Excel and analysis was performed using a custom script provided by Structural Genomics Consortium, University of Oxford. The temperature range over which protein unfolding occurred was established at temperatures below the maximum fluorescence intensity. The processed data were fitted to the Boltzmann equation by non-linear regression analysis using Origin Pro software.

### 2.4 Transcription of Nrf2 targeted genes

Measurements of Nrf2 protein were made with the primary dermal fibroblast cell line 1BR (a gift from Dr Mieran Sethi, King's College London). Cells were grown [26] to ∼ 70% confluency in 12-well plates containing DMEM (Invitrogen, UK) supplemented with 10% (v/v) FCS (Sigma-Aldrich, UK) and 1% (w/v) each of penicillin and streptomycin (Invitrogen, UK). Cells were irradiated with 100 J/cm^2^ of UVA1 (340–400 nm) radiation using a Loctite LED flood array system with peak output at 385 nm and FWHM of 7 nm (Loctite, Henkel Ltd, UK). The spectrum was measured using a DM120BC double monochromator spectroradiometer (Bentham Instruments, Reading, UK) using an integration sphere, calibrated by the Centre for Radiation, Chemical and Environmental Hazards (CRCE), Public Health England against a UK national standard. Irradiance of the sources was routinely measured with a Loctite UVA/Vis radiometer (Loctite, Henkel Ltd, UK). Cells were then treated by the addition to the medium of 100 μM of either porphyra-334 or shinorine, or 10 μM sulforophrane for 24 h. Sulforophane concentration was determined based on maximum concentration found not to induce significant toxicity to the cell line (viability > 75%). All treatments were repeated in triplicate. Total RNA was isolated from the cells using the mirVana miRNA isolation kit with phenol (Life Technologies, USA) on ice according to the manufacturer's instructions. Synthesis of cDNA was carried out using a High Capacity cDNA Reverse Transcription Kit (Applied Biosystems by Life Technologies, USA), again following the manufacturer's instructions. Quantitative RT-PCR of the following genes was performed in 96-well plates using TaqMan Gene Expression Assays (Thermoscientific, Waltham, USA) according to the manufacturer's instructions: *NFE2L2* (encoding human Nrf2), *Keap1*, *GCLC* (glutamate-cysteine ligase), *GCLM* (glutamate-cysteine ligase modifier subunit), *HMOX-1* (heme oxygenase 1), and *MMP-1* (matrix metalloproteinase 1). The amplified products were analysed using an ABI prism 7900 HT sequence detection system (Applied Biosystems). The housekeeping gene *GAPDH* (glyceraldehyde 3-phosphate dehydrogenase) was labelled using 4,7,2′-trichloro-7′-phenyl-6-carboxyfluorescein as a positive control. The probes for the genes of interest were labelled with 6-carboxyfluorescein (Life Technologies, USA). Transcription of *NFE2L2*, *Keap1*, *GCLC*, *GCLM*, *HMOX-1* and *MMP-1* were normalised to that of the *GAPDH* gene. Data analysis was performed using the ΔΔCycles threshold (Ct) method [25]. The ΔΔCt value was determined using the formula: ΔΔCt = (Ct_GOI_ -Ct_HK_), with Ct_GOI_ being the average Ct value for the gene of interest and Ct_HK_ the average Ct value of *GAPDH*. These data were expressed as mRNA expression fold changes, relative to the calibration sample. Assuming a doubling of material during each PCR cycle, the relative quantification (RQ) was estimated according to the formula: (RQ) = 2^−ΔΔCt^.

### 2.5 DPPH radical scavenging activity assay

The percentage of antioxidant scavenging activity for each MAA was determined as follows, each MAA (62.5 μM, 117.7 μM and 210.5 μM concentrations) and an ascorbic acid positive control (3.9 μM, 7.8 μM, 15.6 μM, 31.3 μM, 62.5 μM and 312.5 μM concentrations) were prepared in DMSO. For each sample, the reaction mixture consisted of 0.1 mL of the test sample and 1.5 mL of 70 μM DPPH in methanol. The colour change from violet to yellow, when DPPH is reduced upon reaction with an antioxidant, was recorded at 515 nm using a UV/VIS spectrophotometer (model 7315, Jenway Ltd., Stone, Staffordshire, UK) after 30 min at room temperature, with reaction mixtures shielded from light. The mixture of DMSO (0.1 mL) and DPPH (1.5 mL) served as the reaction blank. The percentage of DPPH radical scavenging activity was calculated as: % *scavenging activity* = *100 x (A*_*blank*_ – *A*_*sample*_*)/A*_*blank*_. The DPPH scavenging activity was proportional to the MAA concentration (for levels examined), and the 515 nm absorbance of the fully reduced DPPH was set to zero. Experiments were performed in technical triplicates with three replicates, and % scavenging activities were plotted as the mean of the 9 triplicate/replicate values against test sample concentrations (μM). IC_50_ values were estimated from linear regression of the data.

### 2.6 ORAC antioxidant assay

The oxygen radical absorbance capacity for each MAA was carried out using the ORAC Antioxidant Assay Kit (Zenbio, North Carolina, USA) according to manufacturer's instructions. Trolox standards were prepared in the assay buffer (0–100 μM) along with serial dilutions of each MAA (500-0 μM) and an ascorbic acid positive control (0–100 μM). 150 μl of the fluorescein working solution was added to central wells of a 96 well plate, with 25 μl of each of the standards or MAA in duplicate, and the plate incubated at 37 °C for at least 15 min. The 2,2′-azobis(2-amidinopropane) dihydrochloride [AAPH; sold as 2,2′-azobis(2-methylpropanimidamine) dihydrochloride] working solution was added to each well (25 μl) to initiate the reaction. Fluorescence was measured in a preheated incubation chamber (37 °C) using a Spectra Max 384 Plus spectrophotometer with excitation/emission = 485/530 nm immediately (t = 0) and then every minute for 30 min. Standard curves were generated for each compound and the area under the curve calculated. Each MAA tested was expressed as a Trolox equivalent concentration.

## 3 Results

### 3.1 MAAs Porphyra-334 and shinorine compete with Keap1-Nrf2 interaction *in vitro*

**Fig. 3:**
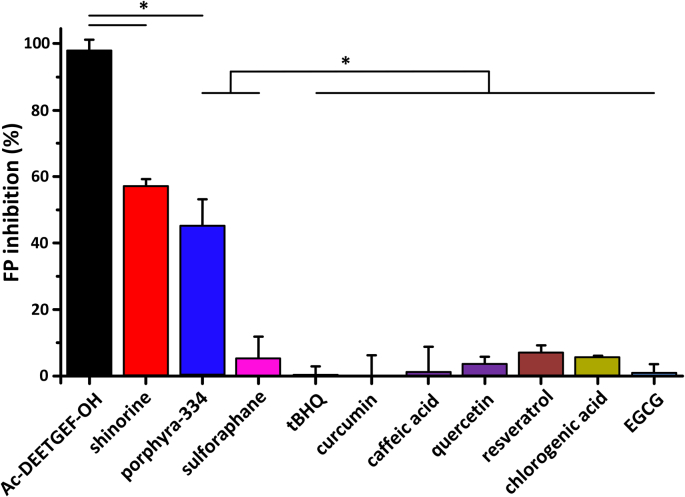
**Measurement of specific, non-reactive binding of MAAs to the Kelch-repeat domain of Keap1.** Ligand-receptor binding was measured using a fluorescence polarization assay in comparison to a high affinity, labelled synthetic peptide. The MAAs porphyra-334 and shinorine gave moderate IC_50_ values of ∼100 μM, whereas no significant interactions were detected between the Keap1 Kelch-repeat domain and any of the eight selected antioxidants or known electrophilic Nrf2 activator sulforaphane. The figure shows results for 100 μM concentration of MAAs and known antioxidants. Error bars represent one standard deviation of the mean, based on 3 experimental replicates, and statistically significant results (based on p-value of two sided *t*-test ≤ 0.05 adjusted for multiple testing based on Bonferroni correction) are marked with an asterisk.

The MAAs porphyra-334 and shinorine together with eight selected antioxidants (ascorbic acid, tBHQ, caffeic acid, chlorogenic acid, curcumin, quercetin, EGCG, trans-resveratrol) plus the known electrophilic Nrf2 activator sulforaphane were evaluated for their interaction with the Kelch-repeat domain of the Keap1 protein using both *in vitro* FP and thermal shift assays. In the FP assay, the test compounds competed with a fluorescein labelled peptide (FITC-[β-Ala]-DEETGEF-OH) based on the high affinity ETGE motif that binds Nrf2 to the Kelch-repeat domain (Fig. 3). The protein-protein interaction of the FITC-[β-Ala]-DEETGEF-OH peptide had a K_D_ of 96 nM in the presence of the Kelch-repeat domain protein and the unlabelled Ac-DEETGEF-OH peptide competed with this interaction with an IC_50_ of 5.4 μM [23]. Both porphyra-334 and shinorine gave nearly equivalent ligand-receptor estimated IC_50_ values of ∼100 μM, requiring a much greater concentration in the presence of excess Kelch-repeat domain protein than the binding of the native peptide sequence [23]. In contrast, no significant interactions were detected between the eight known antioxidants or sulforaphane and the Kelch-repeat domain protein.

The MAAs and eight selected antioxidants were then tested as ligands of the Kelch-repeat domain protein using the thermal shift (differential scanning fluorimetry, DSF) assay. The assays involved treatment of the Kelch-repeat domain protein with the test compounds of interest, followed by heating of the samples to denature the protein. Ligands that bind and stabilise the Kelch-repeat domain protein result in higher denaturation (Tm) temperatures for the complex compared to the free protein. An unlabelled version of the peptide (Ac-DEETGEF-OH) used in the FP assay was used as the positive control in the thermal shift assays against which binding of the test compounds to the Kelch-repeat domain were compared. When used at a 50 μM concentration, the Ac-DEETGEF-OH peptide had a ΔTm relative to the unbound protein of 3.91 ± 0.04 °C. Both porphyra-334 and shinorine also demonstrated elevated ΔTm values to the Kelch-repeat domain protein, albeit when used at 100 μM (porphyra-334 ΔTm = 0.93 ± 0.02 °C, shinorine ΔTm = 0.64 ± 0.11 °C). However, no significant protein-ligand interactions were detectable when the eight antioxidants or the electrophilic Nrf2 activator sulforaphane were tested. The thermal shift assays reflected the results of the FP assays for binding of MAAs and test substrates to the Kelch-repeat domain protein of Keap1.

### 3.2 Porphyra-334 and shinorine induce Nrf2-regulated antioxidant response in fibroblasts

**Fig. 4:**
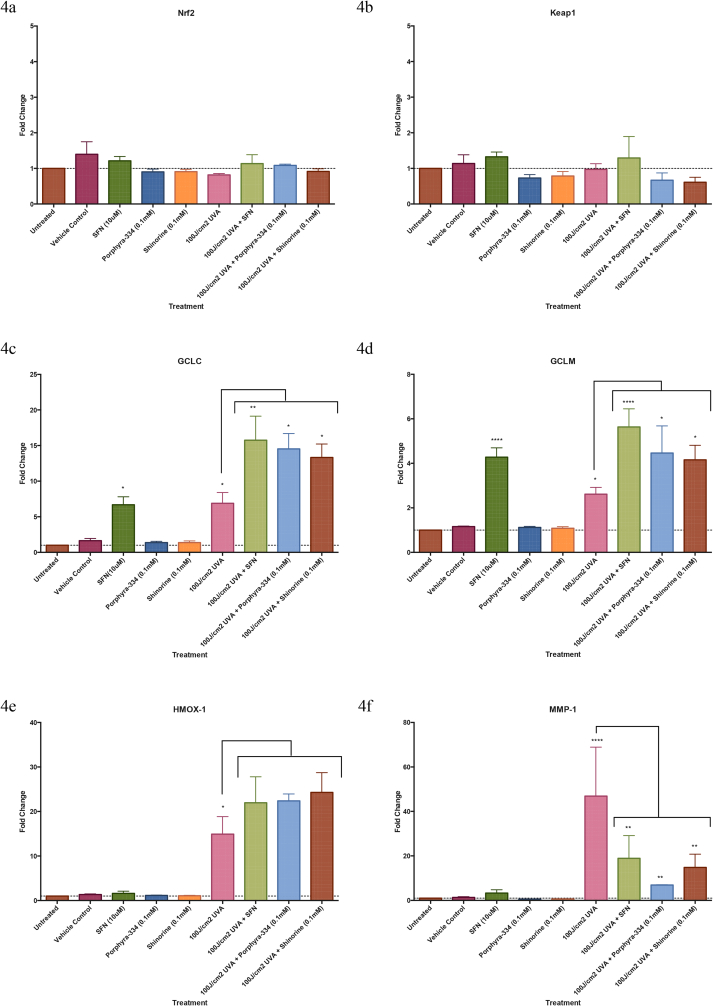
**Transcription of Nrf2 targeted genes.** Differential expression of genes associated with Nrf2 activation: 4a. *NFE2L2*, 4b. *Keap1*, 4c. *GCLC*, 4d. *GCLM*, 4e. *HMOX-1*, 4f. *MMP-1*. Fold changes in transcription were measured ± UVA1 irradiation (100 J/cm^2^) and post exposure incubation when porphyra-334 (100 μM), shinorine (100 μM) or sulforaphane (10 μM) were added to the growth medium. There was no change in the expression of *NFE2L2* or *Keap1* genes with any treatment. UVA1 treatment led to a significant increase in expression for all other genes tested (p < 0.05). Treatment with the MAAs alone led to no significant change, however when cells were irradiated and incubated with MAAs or sulforaphane post exposure there was a significant increase in *GCLC* and *GCLM* expression over that of the cells exposed to UVA1 radiation alone (p < 0.05). A similar relationship was observed for *HMOX-1*, however the data was not significant. Expression of *MMP-1* was significantly increased following UVA1 exposure, however this was significantly reduced following post exposure treatment with all test compounds (p < 0.05). There was no change in *MMP-1* expression with any treatment without UV irradiation. Each column represents the mean value ± SEM (n = 4).

Porphyra-334 and shinorine, together with the known Nrf2 activator sulforaphane were added to primary human dermal fibroblast cells prior to and post UVA irradiation, with UVA dose of 100 J/cm^2^ which is equivalent to a single-day exposure to summer-day sunlight [27]. Gene expression levels for Nrf2, Keap1 and for Nrf2 targeted downstream genes glutamate-cysteine ligase catalytic subunit (GCLC), glutamate-cysteine ligase modifier subunit (GCLM), heme oxygenase 1 (HMOX1) and matrix metalloproteinase 1 (MMP-1) were also measured 24 h after addition of each test compound. The results showed no significant changes in Nrf2 (Fig. 4a) or Keap1 (Fig. 4b) gene expression levels, which was expected for constitutively expressed genes. However, GCLC (Fig. 4c) and GCLM (Fig. 4d) gene expression was significantly enhanced in the presence of MAAs and sulforaphane after UVA exposure. Expression of these genes was also significantly increased in the absence of UVA-induced oxidative stress following addition of sulforaphane, but not following the addition of any of the MAAs without prior UVA treatment (Fig. 4c and d). HMOX1 gene expression had increased also in the presence of these test compounds post irradiation, although this increase was not statistically significant (Fig. 4e). There was a significant increase in MMP-1 gene expression following UVA irradiation, which was significantly reduced following addition of the MAAs and sulforaphane (Fig. 4f). Again, MMP-1 gene expression was only repressed in the presence of MAAs following induction of oxidative stress, whereas gene expression could be significantly reduced upon exposure of fibroblasts to sulforaphane without prior UVA irradiation.

### 3.3 Porphya-334 and shinorine are *in-vitro* antioxidants

Scavenging of the DPPH free radical is a common assay used to assess the antioxidant activity of a compound. Ascorbic acid is a well-known DPPH free-radical scavenger and is commonly used as a positive control, which was used also for comparison in this study. The IC_50_ value for ascorbic acid was 24.5 ± 1.1 μM in the methanol reaction medium. The free radical scavenging activities of MAAs were markedly lower compared to that of ascorbic acid. The IC_50_ values were: 185.2 ± 3.2 μM for porphyra-334 (equivalent to 13.23% of the activity of ascorbic acid) and 399.0 ± 1.1 μM for shinorine (equivalent to 6.14% of the activity of ascorbic acid).

**Fig. 5:**
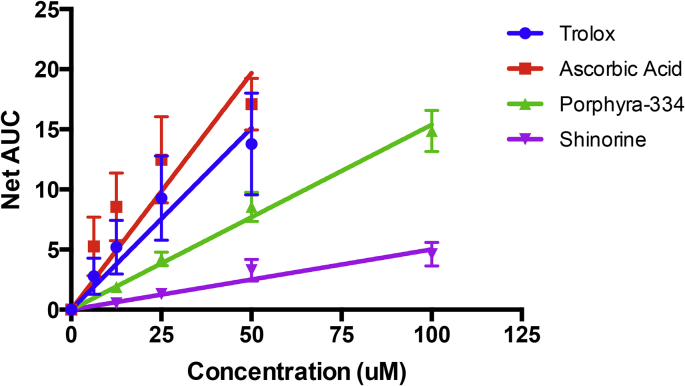
**The oxygen radical absorbance capacity of MAAs.** MAAs and reference controls at increasing concentration were assessed for their ability to quench the [ROO·] radical as a measure of antioxidant activity. All compounds demonstrated significant activity (trolox p = 0.0002; ascorbic acid p = 0.0015; porphyra-334 p < 0.0001; shinorine p = 0.0002; determined by linear regression analysis). Relative antioxidant capacity compared to trolox was calculated (ascorbic acid = 130 ± 12%; porphyra-334 = 51 ± 7%; shinorine = 17 ± 7%). Each data point represents the mean value ± SEM (n = 3).

Another method commonly used to assess antioxidant activity is the ORAC assay, which is used to assess the hydrogen atom transfer capacity of a test compound to suppress peroxyl radical (ROO·) induced oxidative damage measured by the radical-induced fluorescence decay of fluorescein. Trolox, a water-soluble vitamin E analogue, was used as the standard against which other compounds typically are compared, and ascorbic acid was used also as an additional control reference. Both MAAs demonstrated significant activity (Fig. 5) with porphyra-334 displaying an antioxidant capacity equal to 51 ± 7% of equimolar Trolox and shinorine showing 17 ± 7% of the Trolox capacity. Ascorbic acid showed greater antioxidant capacity than did Trolox (equivalent to 130 ± 12% of Trolox) and that of our test MAAs. These controls are shared with previously published work [26].

## 4 Discussion

Aerobic organisms are regularly challenged by various environmental oxidants and toxic electrophiles that include free radicals produced from exposure to UV, visible and IR radiation, xenobiotic pollutants and endogenously generated, reactive by-products of oxidative metabolism. As such, these organisms have long evolved a robust detoxification and redox signalling systems to prevent cellular damage [4,9]. The transcription factor Nrf2, targeting ARE upstream promoter sites of many protective genes, is the principal regulator of mammalian cellular antioxidant defence and P-450 cytochrome detoxification proteins. While various intrinsic pathways and signalling cascades have been identified that contribute to the regulation of Nrf2 function [4,28], the key role of the repressor Keap1 protein is firmly established since disruption of Keap1-Nrf2 binding is sufficient to increase nuclear Nrf2 activity significantly [29,30]. This is supported additionally by siRNA knockdown of Keap1 mRNA [31] and Keap1-null ablation [29], both of which massively enhance free cellular Nrf2 accumulation. Furthermore, somatic mutations of Keap1, a characteristic of certain cancer phenotypes [32,33], result in constitutive Nrf2 activation, as does the silencing of Keap1 expression by miRNAs [34,35], or by epigenetic hyper-methylation of the Keap1 promoter [36].

Human Keap1 contains six ROS/electrophile-sensing cysteine residues (Cys151, Cys226, Cys273, Cys288, Cys434 and Cys613), which are prone to oxidation and covalent modification by electrophiles. These changes do not prevent Keap1-Nrf2 binding, but alter the basal-state “open” conformation, which prevents CRL^Keap1^ ubiquitination to release Keap1 upon Nrf2 degradation [7]. As a result, free Keap1 is not regenerated, and newly synthesised Nrf2 accumulates to promote gene transcription. Electrophile activators of Nrf2 and the majority of other known Nrf2 inducers, including sulphoraphane used in this study, function by covalent targeting critical Cys residues of Keap1 to activate Nrf2 transcription [4,37]. The compounds examined in this study, with the exception of the Keap1-binding MAAs, are sometimes referred to as “indirect antioxidants” or “Nrf2-activating antioxidants” and would not be expected to show appreciable activity in the FP, thermal shift, DPPH or ORAC assays. These expectations were matched by our assay results.

Non-electrophile activators of Nrf2, e.g. small-peptides [23,38] and small-molecule Nrf2 activators [39,40], function by competitive inhibition of Keap1-Nrf2 binding. These compounds hinder the formation of the “closed” Keap1-Nrf2 conformation by binding to the Keap1 β-propeller structure responsible for DLG and ETGE binding. While less studied than electrophile-based Nrf2 activation, targeting of the Keap1-Nrf2 binding site by competitive inhibitors has potential to upregulate Nrf2 activity without the toxic danger of indiscriminate “off-target” effects. These compounds and peptides do not depend on covalent electrophile modification of protein cysteine residues and thus are not prone to indiscriminate reactions with proteins of other cellular signalling networks [2]. In this study we present empirical *in vitro* evidence for the MAAs porphyra-334 and shinorine to competitively interact with the Nrf2 binding site of the human Keap1 protein, as previously anticipated from bioinformatic, structure-based Kelch domain binding predictions [20].

The first study to examine the antioxidant properties of MAAs, using the phosphatidylcholine peroxidation inhibition assay, found that imino-MAAs (shinorine, porphyra-334, palythine, asterina-330 and palythinol) were oxidatively robust to AAPH-generated peroxyl radicals, whereas the oxo-MAA mycosporine-glycine strongly inhibited radical-initiated phosphatidylcholine autoxidation in a concentration-dependent manner [41]. 4-Deoxygadusol, presumed to be the immediate biochemical precursor of MAAs, was found also to have strong antioxidant properties [42] as demonstrated by voltamperogramic comparison of the electrochemical properties of 4-deoxygadusol, mycosporine-glycine and mycosporine-taurine [16]. Numerous studies on the antioxidant properties of MAAs followed thereafter and have been extensively reviewed [43]. Notably, the antioxidant capacities of porphyra-334 and shinorine, together with other MAAs isolated from marine extracts (three seaweeds and one lichen), were compared using the ABTS radical-cation decolorization and superoxide-scavenging pyrogallol autoxidation assays, which showed scarce activity in comparison to mycosporine-glycine [44]. Yet, shinorine and porphyra-334 showed modest antioxidant activity using the β-carotene/linoleate bleaching assay [45]. Contrasting reports by Rastogi et al. attribute significant antioxidant activities of cyanobacterial extracts containing a mixture of imino-MAAs, albeit the IC_50_ values of these MAAs were approximately 15–30% that of the antioxidant activity of ascorbic acid as measured by DPPH-radical, ferric-reducing antioxidant power (FRAP) and superoxide radical scavenging activity (SRSA) assays [45,46]. We report also that purified shinorine and porphyra-334 showed low antioxidant activity in the DPPH radical assay [26] but exhibited significant antioxidant activity using the ORAC assay, which is a measure of the oxygen radical absorbance capacity of a test substance. We recently demonstrated also using the ORAC assay that palythine has significant antioxidant activity having an antioxidant capacity comparable to that of ascorbic acid [26]. Our DPPH assay findings are consistent with established data [43], whereas our ORAC assay results reveals that both pophyra-334 and shinorine have significant antioxidant capacity by quenching oxygen radicals by hydrogen atom transfer, rather than by reductive electron transfer [26].

Several recent publications describe the immunoregulatory and anti-inflammatory properties of mycosporine-glycine, shinorine and porphyra-334 [[47], [48], [49]]. Suh et al. [47] reported that treating the human keratinocyte cell line HaCaT with mycosporine-glycine resulted in a significant decrease in UVR-induced, inflammatory COX-2 mRNA levels, whereas all MAA treatments increased expression levels of the UVR-suppressed, procollagen C proteinase enhancer and elastin genes. Becker et al. [48] found that both shinorine and porphyra-334 induced nuclear factor NF-ĸB activity in the NF-ĸB/AP-1 reporter myelomonocyte cell line THP-1-blue, although NF-ĸB induction was greater with shinorine. Yet, while shinorine marginally enhanced LPS-superinduced NF-ĸB activity, porphyra-334 significantly reduced the NF-ĸB response in LPS-stimulated cells. Ryu et al. [49] had reported previously that porphyra-334 had an inhibitory effect on the expression of NF-ĸB dependent inflammatory genes, such as IL-6 and TNF-ĸB, in UVA-irradiated skin fibroblasts, but pertinent to this study is finding that porphyra-334 can activate the Nrf2 signalling pathway in UVA-irradiated cells. However, nuclear Nrf2 translocation by porphyra-334 without concurrent ROS production by UVA exposure had not been demonstrated [49]. Our gene expression results afforded similar trends to these data [[47], [48], [49]], with enhanced expression of Nrf2 targeted downstream genes following exposure to MAAs or sulforaphane in cells pre-exposed to UVA-induced oxidative stress (Fig. 4c–e). Surprisingly, when cells were treated with MAAs but in the absence of prior oxidative stress, there was no change in expression levels of *GCLC*, *GCLM* or *HMOX-1* genes, yet conversely, the expression levels of these genes were enhanced by treatment with sulforaphane alone. This unexpected observation suggests that MAAs are antagonists of Keap1-Nrf2 binding only after exposure to cellular conditions of oxidative stress, whereas sulforaphane induced Nrf2 activation regardless of the cellular redox state. Previous research has demonstrated that UVA irradiation is insufficient alone to heighten translocation of Nrf2 protein to the nucleus, or to activate expression of Nrf2 targeted genes [50]. Over expression of Nrf2 targeted genes is known, however, to afford enhanced UV-induced photo-protection as demonstrated in a variety of different cell-lines [26,46,48] and a mouse model [51]. UVA irradiation of mammalian cells does enhance the expression of genes regulated by transcription factors YAP and NF-kB [52,53], yet the genes targeted by these transcription factors are distinct from those regulated by Nrf2. Thus, UVA induced activation of YAP and NF-kB cascades is unlikely to explain the over expression of *GCLC*, *GCLM* or *HMOX-1* demonstrated in this study, and also reported in the previous investigation by Ryu et al. [49]. It has been recognised that the activity of Nrf2 can be regulated independently of Keap1 through the NF-kB cascade [[52], [53], [54]] and by the activity of other UV-sensitive proteins such as the small Rho GTPase, Rac1 [55,56]. We suggest that UVA-activated kinases might weaken the interaction between Keap1 and Nrf2, allowing porphyra-334 and shinorine to inhibit Keap1-Nrf2 binding at concentrations insufficient to interrupt these interactions in cells not exposed to UVA. However, the molecular stress-signalling process of this activation is clearly yet unknown and warrants future study, although we posit that enhanced release of Nrf2 from Keap1 by MAAs conditional to UVR-induced oxidative stress could be advantageous for future therapeutic development of these natural products. *MMP-1* gene expression is widely used as an accepted marker of oxidative stress-induced photoageing of human skin [57]. When cells were treated with either the test MAAs or sulforaphane post UVA exposure, there was a significant decrease in *MMP-1* expression, clearly demonstrating a protective effect (Fig. 4f). Again, transcriptional regulation by MAAs was only observed in cells that had been prior irradiated. These results lend support to our findings that MAAs are prospective activators of the cytoprotective Keap1-Nrf2 pathway by Keap1 receptor antagonism. Evidence of nuclear translocation of Nrf2 must now be collected if the ability of porphyra-334 and shinorine to dissociate Nrf2 from Keap1 is to be unambiguously confirmed.

Sustained oxidative damage is a primary cause of inflammation, ageing and contributes to numerous age-related chronic diseases including cancer, diabetes, atherosclerosis, hypertension, stroke and dementia [[58], [59], [60]]. Accordingly, Nrf2 activation has been shown in mouse models to mitigate the progression of various neurodegenerative disorders: Alzheimer's disease, Parkinson's disease, amyotrophic lateral sclerosis, Huntington's disease, and multiple sclerosis [61]. It is widely accepted that oxidative stress increases with ageing [62] due, in part, to a corresponding loss of cellular Nrf2 activity [63]. Suppressed Nrf2 activity is considered a driver of premature ageing [64], and constitutive levels of Nrf2-signalling is claimed to be a key determinant of health span and species longevity [[65], [66], [67]]. Contrary to these benefits, and although low levels of Nrf2 activity predispose cells to chemical carcinogenesis, loss of Keap1 function and elevated Nrf2 levels have been detected in various cancer tumours, which enhances cell proliferation and resistance to chemotherapeutic drugs [[68], [69], [70]], a phenomenon dubbed the “dark side of Nrf2” [71] and the “double-edge sword of Nrf2” [72]. Furthermore, extensive mapping of somatic mutations in human cancer tumours has identified a high number of mutations clustered in the Neh2 domain of Nrf2 within the proximity of the ETGE and DLG motifs [73,74]. Where Nrf2 is constitutively hyper-activated in certain cancer phenotypes, such would render these tumours difficult to treat, leading to poor clinical prognosis [32,75,76], while cancers with low Nrf2 activity appear less resistant to chemotherapy and are thus more successfully treated [77]. Cancers can be classified depending on Nrf2 activity levels, and clinical determination to inhibit Nrf2 activity has potential to aid in cancer treatment [78,79]. The binding affinity of porphyra-334 and shinorine to Keap1 (∼100 μM) were weak in comparison to reported Keap1-Nrf2 PPI activators. For example, Jiang et al. [15], demonstrated that a synthetic compound could effectively disrupt Keap1-Nrf2 PPI with an EC50 of 28.6 nM in the FP assay. Jain et al. [80] similarly reported that the ethyl ester of a synthetic naphthalene-based non-electrophilic compound could also disrupt Keap1-Nrf2 PPI in the nanomolar range, with an IC_50_ of 61 nM in an FP assay. Given consideration that excessive activation of Nrf2 regulated genes may be deleterious, we contend that systemic “soft” Nrf2 activation by the natural products shinorine and porphyra-334, having moderate but specific affinity for binding to Keap1 in specific response to cellular oxidative stress, may be highly desirable [81].

## 5 Conclusion

We demonstrate that pophyra-334 and shinorine, sourced from commercial food-grade seaweeds, are unique natural products that can provide both a direct antioxidant defence and stimulate enhanced cytoprotection by activating the molecular Keap1-Nrf2-ARE pathway. Commercial development of MAAs for use in topical sunscreens has been rekindled with evidence now that certain synthetic organic filters can damage the environment and possibly be harmful to human health [19,26]. Likewise, therapeutic benefits in human degenerative diseases of ageing also wait validation by testing in appropriate disease models.

## Conflicts of interest

The authors declare that they have no conflicts of interest with the contents of this article.

## Author contributions

PFL conceived and supervised the study; GW and KPL designed experiments; KY, GW and ARY provided chemicals and reagents; RG, NGD and KPL performed experiments; all of the authors analysed data, wrote the manuscript and made manuscript revisions.

## Funding

This work was supported by the Medical Research Council UK (grant G82144A). This work was presented in part at the OCC World Congress and Annual SFRR-E Conference 2017 Metabolic Stress and Redox Regulation Berlin, Germany 21–23 June 2017 with an abstract published in *Free Radic. Biol. Med.* **108**, Suppl. 1, page 21.

## Abbreviations

AAPH, 2,2′: Azobis(2-amidinopropane) dihydrochloride
ABTS, 2,2′: azino-bis(3-ethylbenzothiazoline-6-sulphonic acid)
ARE: antioxidant response element
BTB: Broad complex, Tramtrack and Bric-a-Brac domain
caffeic acid: 3-(3,4-Dihydroxyphenyl)-2-propenoic acid
chlorogenic acid: 3-(3,4-dihydroxycinnamoyl)-quinic acid
CRL^Keap1^: Cullin3-Rbx1 E3 ubiquitin ligase – Keap1 complex
Curcumin: (1E,6E)-1,7-bis(4-hydroxy-3-methoxyphenyl)-1,6-heptadiene-3,5-dione
DMSO: Dimethyl sulfoxide
DPBS: Dulbecco's phosphate buffered saline
DPPH: 2,2-diphenyl-1-picrylhydrazyl
ECH: erythroid cell-derived protein with CNC homology
EGCG: Epigallocatechin gallate
FITC: fluorescein isothiocyanate
FP: fluorescence polarization
FRAP: ferric-reducing antioxidant power
FWHM: full width at half maximum
(GCLC): glutamate-cysteine ligase
(GCLM): glutamate-cysteine ligase modifier subunit
(HMOX-1): Heme Oxygenase-1
IVR: intervening region of Keap1
Keap1: Kelch-like ECH-associated protein 1
KR: Kelch repeat
MAAs: Mycosporine-like amino acids
Maf: musculoaponeurotic fibrosarcoma
(MMP-1): matrix metalloproteinase 1
Neh: Nrf2-ECH homology domain
Nrf2: Nuclear factor erythroid 2-related factor 2 protein
ORAC: Oxygen radical absorbance capacity
PPI: protein-protein interaction
Quercetin: 2-(3,4-dihydroxyphenyl)-3,5,7-trihydroxy-4*H*-chromen-4-one
Rbx1: cullin-RING-based E3 ubiquitin-protein ligase, encoded by RBX1 gene
SRSA: superoxide radical scavenging activity
trans-resveratrol: 3,5,4′-trihydroxy-trans-stilbene
sulforaphane: 1-Isothiocyanato-4-methylsulfinylbutane
Tbhq: tert-butylhydroquinone
